# Setting up the first human milk bank in Uganda: a success story from Nsambya hospital

**DOI:** 10.3389/fnut.2023.1275877

**Published:** 2024-01-10

**Authors:** Victoria Nakibuuka, Janat Kainza, Ritah Nasiima, Sanyu Nalunga, Ritah Nazziwa, Hamim Mponye, Christinah Nuwahereza, Ronald Kyambadde, Racheal Nantenza, Caroline Nassonko, Barbara Nalubwama, Immaculate Nabwami, Madrine Nabaliira, Cleophas Kabategweta, Olivia Nalule, Joannita Nampijja, Barbara Namugga, Peter Kirabira, Gillian Weaver

**Affiliations:** ^1^St. Francis Hospital Nsambya, Kampala, Uganda; ^2^Lubaga Hospital, Kampala, Uganda; ^3^Mengo Hospital, Kampala, Uganda; ^4^International Human Milk Banking Specialist and Consultant, Human Milk Foundation, Harpenden, United Kingdom

**Keywords:** breastfeeding, milk banking, milk bank, human milk, lactation

## Abstract

**Background:**

The World Health Organization (WHO) strongly recommends the use of donor human milk (DHM) for low birth weight infants when mother’s own milk is unavailable or insufficient. However, the use of DHM requires the availability of human milk banks (HMBs), the majority of which are in middle and high-income countries. Developing countries offer multiple opportunities and challenges regarding the establishment and operationalization of HMBs. This study describes the experience in setting up the HMB in Uganda at St. Francis Hospital Nsambya.

**Methods:**

The establishment of the first HMB in Uganda followed a step-wise approach using the PATH’s Resource Toolkit for Establishing and Integrating Human Milk Banks. The steps included: performing a facility readiness assessment, implementing quality control measures, forming and training a committee for the Baby Friendly Hospital Initiative, establishing a monitoring and evaluation system, developing a communication strategy, engaging with the Ministry of Health, providing staff training by a Human Milk Bank consultant, and maintaining regular coordination by a dedicated technical team.

**Results:**

A total of 170 donors have been screened and of these 140 have donated milk with a mean age of 26 years since the establishment of the bank in November 2021. A total of 108 admitted neonates have received the milk; majority (88%) are preterm infants with a mean gestational age of 34 weeks. A total of 90 liters have been collected and 76 distributed. The challenges in establishment of the Human Milk bank included: lack of guidelines on human milk banking, use of unpasteurized milk, lack of communication strategy, lack of clear model infrastructure and lactation training. We addressed the challenges: by drafting guidelines, set up a human milk bank and had training on use donor pasteurized milk, designed communication messages through videos and brochures, visited Pumwani hospital and remodeled the Human Milk Bank according to the model at Pumwani, all the health workers in the human milk bank had a training on Lactation. Assessing the experiences and attitudes of mothers, donors, healthcare providers, and hospital leaders revealed concerns about milk safety and fear about potential attachments or acquired traits through the donated milk to the babies that may receive it. Donors viewed milk donation as a life-saving act, although fears of breast cancer and lumps arose from misconceptions. To address these perspectives, creative media, such as videos and messages, were designed to raise awareness, promote behavioral change, and create demand for the HMB services.

**Conclusion:**

The establishment and integration of HMB services at hospitals in Uganda is feasible.

## Introduction

Globally, about 20% of all births, an estimate of more than 20 million infants are born with a low birth weight (LBW) making them 20 times more likely to die that their counterparts (1). Developing countries are disproportionately affected accounting for the majority of LWB babies and highest infant mortality rates in the world (1). In Uganda, LBW babies and premature deliveries account for 1 in 10 deliveries but both accounting for about 3 in 10 infant deaths (2). To avert the high mortality, the World Health Organization (WHO) recommends prioritization of early initiation of breastfeeding for preterm and LBW infants (3). Human milk contains bioactive components that help to protect the medically fragile infant from the development of complications such as sepsis, retinopathy of prematurity, and necrotizing enterocolitis (4, 5). This helps to avert infant mortality, 40% of which occurs within the first month of birth (1). However, the provision of human milk through breastfeeding from mother-to-child is not always a practical option. Many babies may not receive breast milk in the first 48 h of life due to a lack of breast milk, inappropriate lactation and mother’s morbidity or mortality (6, 7). In this event, WHO recommends the provision of pasteurized donor human milk (PDHM) from a human milk bank (HMB), as superior alternative to infant formula or any other breastmilk substitutes (3). The establishment of HMBs has gained increasing recognition as a vital intervention for promoting optimal infant nutrition, and health in complex situations (8). Human breast milk is considered the gold standard for infant nutrition as providing essential nutrients, antibodies, and growth factors that support their healthy development (9).

HMBs are a critical as they facilitate the collection, processing, and distribution of donor human milk and also strengthen breast feeding practices as well (10). They act as a vital link to appropriate infant feeding by providing a safe and reliable supply of donated human milk to infants who cannot access their mother’s milk. These banks ensure that infants receive the benefits of human milk, promoting optimal nutrition, supporting healthy development, and reducing the risks associated with formula feeding (4, 11). They also offer support to mothers who are unable to breastfeed, fostering a sense of community and promoting knowledge about proper breast feeding practices. However, in many parts of the world, access to safe and sufficient breast milk is a significant challenge, especially for vulnerable infants born prematurely or with medical complications (12, 13). This can lead to serious health problems for babies, including poor postnatal growth, infection, developmental delay and even death (5, 14, 15). The absence of HMBs in these regions poses a significant barrier to providing essential, lifesaving breast milk to infants in need. Without access to a reliable and safe source of donor milk, healthcare providers are often compelled to resort to the use of infant formula and breast milk substitutes as alternative feeding options. Unfortunately, this reliance on artificial substitutes can have detrimental effects on both the initiation and sustenance of breastfeeding practices (3, 16–19). The lack of accessible and affordable donor breast milk through HMBs only exacerbates this issue, as it limits the availability of a vital resource that can be instrumental in overcoming breastfeeding challenges.

In 2021, the first human HMB in Uganda was established at St. Francis Hospital Nsambya in Kampala. The milk bank aims to provide a safe and reliable source of breast milk for babies who are unable to get it from their mothers for the first few days until they attain a regular milk supply with adequate lactation support. St. Francis Hospital Nsambya admits about 400 preterm infants annually of which 60 to 70% of these have very low birth weight and are at higher risk of poor postnatal growth, development and death attributable to lack of breast milk (4, 11, 14, 20). Therefore, the establishment of the first HMB in Uganda is a significant milestone in improving survival for preterm infants. This qualitative study aims to explore the process and experiences of initiating the first human breast milk bank in Uganda. The study findings of this study provide valuable insights into the experience of establishing the first HMB in Uganda. By sharing our journey, challenges, and successes in setting up the HMB, this study contributes to the existing body of knowledge on HMB establishment in low-resource settings. The findings highlight the importance of careful planning, collaboration with stakeholders, and the provision of high-quality training and mentorship to overcome the unique challenges faced in such settings. The establishment of the HMB in Uganda serves as a significant milestone in improving the health and well-being of newborns, and it offers a blueprint for other regions to follow in implementing similar initiatives. This study emphasizes the feasibility and potential impact of establishing HMBs in low-resource settings, ultimately aiming to save and improve the lives of vulnerable infants.

## Setting up the first HMB in Uganda

### Location

This study was conducted at St. Francis Hospital Nsambya, a private not-for- profit hospital in Kampala, Uganda, which serves as a tertiary referral centre for Makindye division with a total population of 398,800. The following services are available: obstetrics, paediatrics, internal medicine and surgery. Each of the departments have specialists and medical officers and nurses. It conducts 4,000 deliveries per year and provides 24 h comprehensive emergency obstetric and neonatal care services. The obstetric department has 9 obstetricians, 12 postgraduate doctors and 40 midwives. The neonatal unit admits 2,500 neonates annually, has a bed capacity of 50 and 1 neonatologists, 1 pediatrician, 2 medical officers, 25 nurses and 7 postgraduate students. The unit is currently divided into three levels: (1) Neonatal intensive care unit, (2) Neonatal observation unit, and (3) Kangaroo and isolation unit. The neonatal intensive care unit was opened in 2015 and includes 10 beds with access to oxygen therapy, artificial surfactant, low-cost bubble CPAP and mechanical ventilation. The neonatal observation unit opened in 2006 and has a total capacity of 30 beds with access to phototherapy and bubble CPAP. Together, the kangaroo and isolation unit have a total capacity of 10 beds. The feeding protocol ensures that all preterm infants less than 32 weeks or less than or those that sick are started on trophic feeds at 24mls/kg/day and these are increased by 24mls per kilo day till full freed attainment of 160mls/kg/day. The infants above 1.5Kgs and above and are stable are initiated on breast feeding or Cup feeds or Nasogastric feeds with breast milk at 60mls/kg/day. The nurses on the ward offer lactation support to the mothers to ensure that they have enough breast milk through education and counseling and assisting them to express breast milk on the Ward. Additionally, kangaroo mother care is encouraged for all mother’s even those whose babies are on Bubble CPAP. Overall, the selection of St. Francis Hospital Nsambya as the site for the HMB takes into account the hospital’s role as a tertiary referral centre, its well-equipped neonatal unit, and its existing support structures for breastfeeding and lactation. These factors collectively contribute to the successful implementation and functioning of the HMB, ultimately improving the care and well-being of newborns in the hospital’s catchment area.

### Setting-up process

#### Step 1: Learning visit and document review (January 2019)

A team consisting of a neonatologist, nurse, and technician visited Pumwani Hospital, the first HMB in Kenya (21). The purpose of the visit was to learn about the establishment of HMBs, challenges, lessons learned, and the processes involved. Due to the lack of established Ugandan guidelines on HMB establishment, the team relied on guidance provided by the Pumwani Hospital HMB and an international HMB expert and experts from PATH. All HMB processes were shared, including quality control, collection, screening, storage, pasteurization, prioritization, appropriate use of PDHM in the neonatal unit, donor recruitment, and lactation support. The international HMB technical expert and experts from PATH continued providing mentorship through remote support. The team reviewed project documents and made modifications to the project plan based on what they learned at Pumwani Hospital. They also identified lessons learned throughout the project, such as the importance of early initiation of breastfeeding, milk expression, and frequent expression for building maternal milk supply. The information and guidance received from Pumwani Hospital and the international HMB expert was essential for the successful establishment of a HMB in Uganda. Overall, the learning visits and document review at Pumwani Hospital played a critical role in laying the foundation for the successful establishment of the HMB in Uganda. It provided the team with essential knowledge, guidance, and practical insights from an established HMB, enabling them to adapt and apply these learnings within the unique context of Uganda. The collaboration with experienced professionals and the lessons learned from the visit were instrumental in ensuring the effective implementation of the HMB project in Uganda.

#### Step 2: PATH toolkit adaptation (January 2020)

The PATH HMB Toolkit is an essential resource for anyone involved in the establishment or operation of a human milk bank (22). It is a comprehensive, evidence-based resource that can help organizations ensure that their HMBs are safe, effective, and sustainable. It provides comprehensive guidance on all aspects of HMB operations and is freely. Using the toolkit as a framework, a facility assessment readiness was conducted to assess the existing infrastructure and resources within the newborn unit, which would be transformed to accommodate the HMB. This assessment aimed to identify the strengths and weaknesses of the facility to inform the remodeling process. By adapting the PATH HMB Toolkit and conducting HMB-specific facility assessment readiness, the team gained a thorough understanding of the specific strengths and weaknesses of the facility. This information guided the remodeling efforts to create a suitable and functional space for the HMB. The adaptation of the toolkit and the facility assessment were crucial steps in ensuring that the establishment of the first HMB in Uganda was well-planned, aligned with best practices, and tailored to the local context. The facility assessment enable the team to leverage the strength and mitigate the weakness:

### Leveraging strengths

Overall, leveraging strengths such as current feeding practices, the recognition of the need for donor human milk, the presence of local leadership, existing quality control systems, and building networks with nearby hospitals, was essential for establishing a successful and sustainable HMB. By building upon these strengths, the HMB was able to effectively support breastfeeding, provide safe donor human milk to infants in need, and create a supportive network of healthcare providers and facilities. The hospital was able to overcome many of the challenges that could have arisen.

- *Current Feeding practices:* Alternative feeds were used, including expressing milk for preterm infants and milk sharing between mothers. These practices indicated the willingness and ability of mothers to provide breast milk for their babies, even in challenging circumstances. Leveraging this strength is crucial for promoting and supporting breastfeeding, as well as creating a culture of milk sharing and donation within the community.
- *Identifying need for donor human milk:* Adequate and timely help provided to new mothers to collect their colostrum and initiate lactation when their infants were unable to breastfeed. Health workers also encouraged mothers to do skin-to-skin contact and stay with their babies. This indicated a proactive approach to supporting breastfeeding and encouraging maternal involvement in infant care. Leveraging this support helped facilitate the acceptance and utilization of donor human milk among mothers and healthcare providers.
- *Identifying local leadership:* The presence of a neonatologist, head of the neonatal unit, who was identified as the leader and local champion to establish the HMB. A multidisciplinary leadership team was also identified, consisting of the administrator, the in-charge of the neonatal unit, a microbiologist, a nutritionist, and pediatricians working at the hospital. These leaders possess the necessary expertise, knowledge, and influence to drive the implementation of the HMB and ensure its successful integration within the hospital and the wider healthcare system.
- *Quality Control Systems:* The hospital facility had available appropriate hospital cleaning, disinfection, and equipment maintenance services that were necessary once the human milk bank was established. Leveraging these existing quality control systems ensured that the necessary infrastructure and processes are already established, reducing the burden of implementation and facilitating the smooth functioning of the HMB.
- *Building Networks:* Identified HMB sites to serve as scaling satellite lactation centers, such as the nearby hospitals of Lubaga, Mengo, Kibuli and Naguru. These existing facilities allowed for the establishment of a broader network of support, collaboration, and knowledge sharing, ultimately enhancing the effectiveness and sustainability of the HMB initiative. We anticipate to scale up the Human Milk banking activities to these units in Future.

### Mitigating our weaknesses

The overall importance of mitigating weaknesses in the establishment of an HMB cannot be overstated. By taking steps to address these weaknesses, the hospital was able to ensure that the HMB was established in a safe and effective manner. The initiatives aimed at improving feeding practices, establishing a safe and sustainable supply of donor human milk, engaging stakeholders, ensuring quality control, and fostering collaboration and knowledge sharing. These efforts collectively contribute to improving infant health outcomes, promoting breastfeeding, and addressing the healthcare needs of the community.

- *Current Feeding practices:* Alternative feeds involved the use of unpasteurized milk highlighting the importance of starting a HMB. This addressed the need for appropriate feeding practices and emphasized the importance of promoting donor human milk as an alternative bridge when breastfeeding is not immediately feasible. All the health workers were also trained on the use and safety of pasteurized human milk.
- *Identifying need for donor human milk:* The hospital lacked of well-trained lactation support staff, nurses being too busy to assist with breastfeeding, insufficient privacy and facilities for expressing milk, limited availability of breast pumps, inadequate education on expressing milk, storage facilities, and a lack of counseling and support regarding donor breast milk for mothers. Efforts were made to train all health workers on lactation support and to provide comfortable rooms with privacy for expression with in the human milk Bank premises. These measures aimed to improve breastfeeding practices and create a conducive environment for both donor milk and maternal milk expression.
- *Identifying pool of donors:* The lack of HMB-related infant feeding guidelines made it difficult to establish and use donor human milk. The development of infant feeding guidelines were essential steps toward establishing processes for the identification of donors and utilizing donor human milk effectively. This ensures that there is a reliable supply of safe donor milk and promotes the implementation of evidence-based guidelines for infant feeding.
- *Identifying local leadership:* The establishment of the HMB required involvement of stakeholders beyond the hospital. Approval was obtained from the hospital and regional/national leaders for the integration of the HMB. Potential religious and cultural barriers related to the collection, donation, and acceptance of donor milk were identified or examined. A series of important meetings took place to explore the establishment of a HMB. Initially, a stakeholder meeting was convened between St. Francis Hospital Nsambya and ELMA to discuss the potential of having a HMB. Following this, another meeting was held involving St. Francis Hospital Nsambya, the Ministry of Health (MOH) Assistant Commissioner for Child Health, and the Assistant Commissioner for Nutrition, and other hospitals such as Lubaga Hospital and Mengo Hospital, to discuss the scaling of the HMB initiative. The engagement of the local champion played a key role in these discussions. To ensure effective implementation, an advisory committee consisting of 15 members was carefully selected. This committee held regular meetings to assess local needs, provide guidance, and oversee the implementation of the HMB. Additionally, a Technical Working Group was formed, comprising the champion, three local pediatricians, a microbiologist, ward in-charges, ward assistants, and the hospital engineer. This group was responsible for the day-to-day activities involved in setting up the HMB. The formation of advisory committees and technical working groups allowed for collaborative decision-making, guidance, and oversight in setting up and operating the HMB.
- *Quality Control Systems:* A plan for quality assurance, such as hazard analysis and critical control point (HACCP) and HACCP training, was not in place and this was installed after the training using the PATH tool kit and the international Human Milk Banking expert.
- *Building Networks:* No communication platforms were identified to facilitate the sharing of information and best practices between the learning sites established. Therefore, we are in the process of improving networks with the nearby hospitals. Scaling up of the networks and communication networks will be done subsequently in the years 2023–2025.

#### Step 3: Ensuring quality assurance control (January–December 2020)

First, a Consultant with expertise in HMB was hired in collaboration with PATH to provide guidance and support. With the Consultant’s guidance, the HMB guidelines were developed along with the involvement of a neonatologist from Kenya with expertise in the field. These guidelines covered various components such as infant and child mortality, the importance of breast milk as a life-saving intervention, the process of establishing a Human Milk Bank, handling and processing of the milk, criteria for selecting recipients of donor milk, quality control measures, and monitoring and evaluation protocols. The development of these guidelines involved consultations with the advisory committee of the Human Milk Bank, the Ministry of Health, and the Nutritional departments of Uganda. Following the completion of the guidelines, standard operating procedures (SOPs) and registers were drafted to ensure proper execution of activities within the bank. These included procedures related to donor milk culture, identification, tracking, and tracing, as well as allocation and prioritization of human milk. The registers included various records such as equipment cleaning checklists, stock book data dictionary, stock book for the milk bank, pasteurization log, informed consent for receipt of donor human milk, register of human milk donors, thawing and pooling log, and donor milk daily order forms.

To ensure proper utilization of the SOPs and registers, training was provided to all health workers involved in the Human Milk Banking. Additionally, a workshop on Hazard Analysis Critical Control Points (HACCP) Plan, Lactation support was conducted in collaboration with a lactation consultant and the use of the PATH tool kit. The HMB team received training on the HACCP process, which is a quality assurance planning process for food systems. By utilizing standard guidelines for Human Milk Banking, a HACCP plan was developed, identifying critical control points and interventions to reduce hazards. This process highlighted areas where system strengthening was required to ensure optimal quality and safety within the local context. To promote a holistic approach, a Baby Friendly Hospital Initiative Committee was formed to enhance breastfeeding support and practices within the facility. Additionally, monitoring and evaluation activities were implemented to track progress and measure outcomes. An advisory committee was created to provide expert advice and guidance on the operations of the Human Milk Bank. Overall, the importance of these initiatives lay in creating a well-structured, standardized, and safe HMB that adheres to best practices, guidelines, and quality control measures. By establishing clear protocols, providing training, and engaging with expert advisors, the HMB can effectively support breastfeeding, provide safe donor human milk, and contribute to improving infant health outcomes within the facility and the broader community (Table 1).

**Table 1:** List of registers and standard operating procedures.

Register/SOP	HMB	Neonatal unit
Donor milk culture	√	
Human milk identification, tracking and tracing	√	
Allocation and prioritization of human milk	√	
Cleaning of human milk bank equipment	√	
Discarding of donor milk	√	
Donor screening	√	
Donor recruitment	√	
Handling and storage of donor human milk	√	
Milk expression	√	
Processing and testing of donor human milk	√	
Transportation of donor human milk	√	
Donor screening register	√	
Donor screening questionnaire	√	
Donor informed consent form	√	
Register of human milk donors	√	
Received donor milk log	√	
Thawing and pooling log	√	
Pasteurization log	√	
Human milk stock book	√	
Informed consent for receipt of donor human milk		√
Donated human milk daily order form		√
Donor milk dispensing register	√	
Recipient donor human milk log		√
Donor milk recipient record		√
Human milk bank equipment cleaning checklist	√	

#### Step 4: Procurement, installation, training, and mentorship (January 2021–October 2022)

This period involved the procurement of the necessary equipment, training, and mentorship of the health workers on the use of HMB and lactation. Under the guidance of the HMB consultant and experts from PATH, the facility requirements for the HMB were established by benchmarking them against Pumwani Hospital HMB in Kenya. The hospital administration, along with the HMB team, supervised the construction process. ELMA philanthropies and St. Francis Hospital Nsambya facilitated the procurement, importation, and installation of essential equipment and supplies, including the human milk automated pasteurizer Sterifeed, pasteurization bottles, autoclave, freezers, and refrigerators. A suitable space near the Kangaroo Mother Care room was identified to accommodate the Human Milk Bank, ensuring integration within the health facility. The suppliers of the equipment provided guidance on the proper functioning and maintenance requirements, which would be monitored locally to ensure smooth operations of the HMB. Table 2 shows the cost of equipment. The most expensive item on the list was the pasteurizer, which was estimated to cost 27,078 USD. The other major expenses were the refrigerators (12,997 USD), freezers (19,496 USD), and infrastructure remodeling (23,016 USD). The total estimated cost of procuring the items required for setting up a HMB was 96,035 USD. The initial cost of setting up the HMB was significant, but it is important to note that this was just the one-time cost of purchasing the equipment and supplies needed to start the HMB. The ongoing costs of operating the HMB, such as the cost of staff salaries, are much lower. The majority of the items listed in the table are one-off items, meaning that they were only needed to be purchased once. This includes the pasteurizer, refrigerators, freezers, autoclaves, washing machine, and sterile glass bottles. The infrastructure remodeling cost is also a one-off cost. The only ongoing costs that was incurred after the HMB is set up are the cost of staff salaries (not included in the table), the cost of milk testing, and the cost of consumables, such as gloves, masks, and gowns. These costs are much lower than the initial cost of setting up the HMB. For example, the cost of staff salaries depends on the number of staff employed by the HMB and their salaries yet these salaries can be offset by integration of the HMB responsibilities within existing staff. The Health Workers working were included on the hospital pay roll as well moreover, these some cost can be off-set by applying affordable patient fees for the service. The cost of milk testing depends on the number of milk samples that need to be tested and the cost of the tests. The cost of consumables also depends on the number of consumables used and their cost (22).

**Table 2:** Cost of items procured for the HBM.

Item	Estimated cost (Ugx)	Estimated cost (USD) (1$ = 3,693 Ugx)
Pasteurizer	100,000,000	27,078.26
Refrigerators (2)	48,000,000	12,997.56
Freezers	72,000,000	19,496.34
Autoclaves	5,000,000	1,353.91
Washing machine	3,000,000	812.35
Sterile Glass bottles	3,000,000	812.35
Infrastructure remodeling	85,000,000	23,016.52
Training and Stationary used	500,000	135.39
Consultant Human Milk Banking	28,157,322	7,624.51
Training and stationary used	10,000,000	2707.83
**Overall cost**	**354,657,322**	**96,035**

#### Step 5: Launch (November 2021)

The St. Francis Hospital Nsambya HMB, the first HMB in Uganda encompassing activities such as the collection, processing, storage, and allocation of Pasteurized Donor Human Milk (PDHM) to the neonatal unit. A highly visible official opening ceremony was organized to highlight the crucial role of the HMB in saving newborn lives across Uganda. The MoH officials, Commissioner for Clinical Services, Commissioner for Child Health, Academia including the school of Public Health and many stakeholders will invited for the launch. To create awareness and encourage the use of PDHM, donor recruitment, and breastfeeding promotion, a range of demand generation activities were implemented, including events, one-on-one engagements, mass media campaigns, and social networking. To ensure the smooth functioning of the Human Milk Bank, a nurse from Pumwani Hospital, who is experienced in HMB procedures, was identified to mentor the HMB nurses. Additionally, a systematic approach was taken to recruit donor mothers. A questionnaire was administered to screen potential donors based on age, health status, and their willingness to contribute milk. The establishment of the St. Francis Hospital Nsambya HMB as the first HMB in Uganda represented a significant milestone in improving newborn care and saving lives.

### Operationalization of the human milk bank

A nurse from Pumwani Hospital’s HMB was chosen as a mentor to provide guidance to the HMB Nurses regarding the necessary procedures. The mentor nurse assisted in training the HMB Nurses on how to utilize the registers and adhere to the SOPs that were developed. Additionally, the mentor nurse provided guidance on offering lactation support to mothers. The next phase of the process involved identifying and recruiting mothers who had an excess supply of human milk. A questionnaire was administered to assess their eligibility as milk donors, considering factors such as age, health status, and willingness to participate. The mentor nurse from Pumwani Hospital provided guidance throughout this process. Once a sufficient quantity of milk had been accumulated, the first batch underwent pasteurization, a crucial step to ensure the safety of the donated milk. The mentor nurse provided guidance during the pasteurization process, and samples were collected and sent to the laboratory for further analysis. Overall, the mentor nurse was important in guiding and supporting the HMB Nurses in various aspects, including using registers, following SOPs, providing lactation support, and ensuring the proper collection and processing of donated milk. Their expertise and guidance contribute to the successful establishment and operation of the HMB.

### Collection and recipients of milk

**Figure 1:**
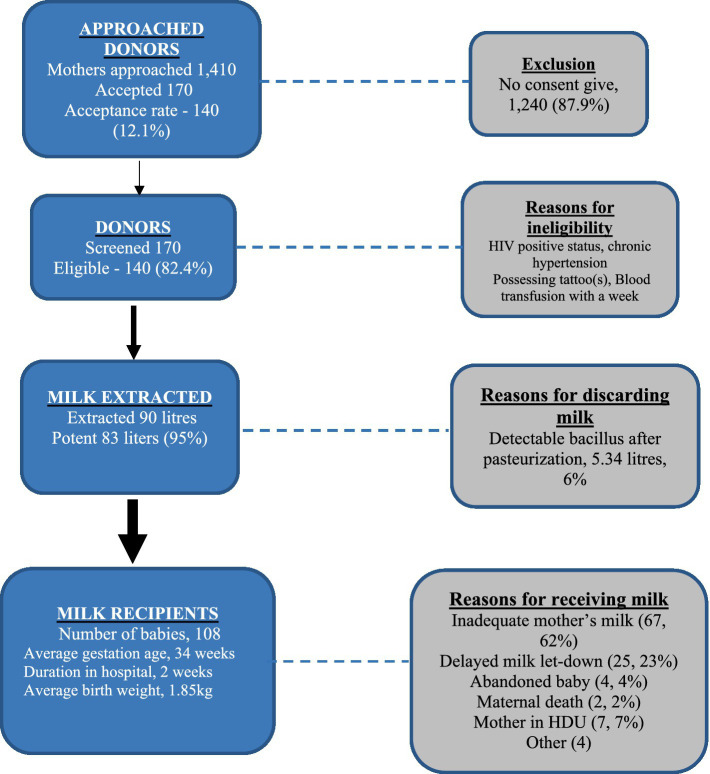
Donor recruitment, milk collection, and milk administration.

Figure 1 shows the donor recruitment, milk collection and milk administration results from the first 10 months of the operation of the St. Francis Hospital Nsambya HMB. During the period, 170 mothers agreed to become donors. Of the donors, 140 were found eligible and subsequently donated milk to the HMB. The average age of the donors was 27 years and 66% of them gave birth St. Francis Hospital Nsambya. Overall, 89 liters of donor milk were collected, 95% of which passed the post pasteurization screening. During the period, the HMB served 108 infants and 72% of whom were preterm with a mean gestation age of 32 weeks. Inadequate mother’s milk was the most common reason for the issuing of donor milk.

### Perspectives regarding collection and use of human milk

A quick comprehensive assessment was conducted to explore the experiences, attitudes, and perceptions of various stakeholders involved in the HMB. The aim was to gather valuable insights to inform the planning, design of a behavioral change communication strategy, implementation of a demand generation strategy, and facilitate future scalability of the human milk bank. Qualitative methods, including focus group discussions and key informant interviews, were employed to gather data. The assessment revealed several noteworthy findings. Mothers whose babies received donated milk expressed concerns regarding the safety of the milk, particularly among those who had not received proper orientation prior to enrollment. Some mothers also expressed worries about their babies developing attachments to the donors or acquiring characteristics from them. These concerns highlight the importance of providing adequate information and reassurance to mothers about the safety and benefits of donated milk to alleviate their anxieties.

*“how can I give my children some else’ milk, I did not know I had only one week given so after delivery the nurses told me that I had to get a donor, I said aaaahh a donor? How am I going to bond with my kids if am going to give some else’ milk?” – A recipient’s mother.*

On the other hand, the donors perceived milk donation as a way to save lives and expressed happiness in knowing that they were contributing to someone else’s well-being. This positive perspective among donors emphasizes the altruistic nature of milk donation and the potential for creating a supportive community of donors.

*“For me when I gave birth to my baby, I delivered by caesarian section. On day 3, I had the…… the milk which I had wasn’t too much but my neighbor had twins and she could suffer with milk so I was advised also to donate the little I had to share with her. This made me feel good because I was saving a life”* – A Milk donor.

Mothers also expressed concerns about their own well-being, with some expressing fear of developing breast cancer and breast lumps as a result of continued milk donation. This highlights the need for comprehensive education and support for donors to address their concerns and ensure their physical and emotional well-being throughout the donation process.

*“I told my mother about breast milk donation, do you know what she told me, she told me that “You, are you serious? Do you know what you are going to acquire? You are going to get cancer, better get serious. Do you know the machines they are using? She also told me that the electricity will enter into me…… Let me stop but on the way after donating, I felt things like a ball moving around in my breast so when I went back and told her that something was moving around in my breast, do you know what she told me, “You see, I told you not to go back there, now you have got cancer”* – A Milk donor.

Overall, the findings underscored the need for effective communication strategies to address the concerns and anxieties of both recipient mothers and milk donors. Providing accurate information, addressing misconceptions, and offering comprehensive support helped alleviate worries and promote a positive environment within the HMB. It is crucial to prioritize the well-being of both donors and recipients and ensure that they are well-informed and supported throughout their participation in the milk donation program. Based on these findings, the research team designed creative media, including short video clips and messages, to raise awareness and sensitize the community about the human milk bank. The aim is to create social behavioral change and generate demand for donated milk. These communication strategies can play a crucial role in dispelling misconceptions, addressing concerns, and promoting the benefits and safety of human milk donation. These findings provide valuable insights for the development and implementation of strategies to enhance the acceptance, utilization, and scalability of human milk banks. By addressing concerns, providing education, and creating awareness in the community, it is possible to foster a supportive environment that promotes milk donation and ensures the well-being of both recipients and donors, ensuring the successful implementation and scalability of the HMB.

## Conclusion

The establishment of the first HMB in at St. Francis Nsambya Hospital in Uganda demonstrates a comprehensive and feasible approach to improving newborn health through the provision of safe and donor human milk. The feasibility of the project is evident in several key aspects. The initial learning visits to Pumwani Hospital in Kenya provided valuable insights and lessons that were adapted to the local context. The utilization of the PATH HMB Toolkit (1) further facilitated the understanding and implementation of best practices in HMB operations. The identification of local leadership, formation of multidisciplinary teams, and engagement of key stakeholders, including the hospital administration, MoH officials, and other hospitals, ensured a collaborative approach to the establishment of the HMB. This widespread involvement and support were instrumental in overcoming potential religious, cultural, and operational barriers. The development of guidelines, standard operating procedures, and a HACCP plan ensured the implementation of robust quality control measures. Training programs provided to health workers, including lactation support training, further enhanced the safety and effectiveness of the HMB operations. The successful procurement, importation, and installation of necessary equipment and supplies, as well as the identification of suitable space within the hospital, showcased the project’s feasibility in terms of resource allocation and infrastructure development. The implementation of demand generation activities, including mass media campaigns, one-on-one engagements, and community sensitization, contributed to the recruitment of donors and the promotion of PDHM usage. The findings from the stakeholder perspectives highlighted the need for comprehensive education and support for both donors and recipients, which can be addressed to further enhance the feasibility and acceptance of the HMB. Overall, the establishment of the HMB at St. Francis Nsambya Hospital in Uganda demonstrates a feasible and sustainable approach, with careful consideration given to local contexts, stakeholder engagement, quality control, infrastructure development, and demand generation. This comprehensive and collaborative effort ensures that safe and donor human milk is available for infants in need, ultimately improving newborn health outcomes in Uganda.

## Data availability statement

The original contributions presented in the study are included in the article/supplementary material, further inquiries can be directed to the corresponding author.

## Ethics statement

The studies involving humans were approved by Nsambya Hospital institutional review board (SFHN-2023-106). The studies were conducted in accordance with the local legislation and institutional requirements. The participants provided their written informed consent to participate in this study.

## Author contributions

VN: Conceptualization, Formal analysis, Funding acquisition, Methodology, Project administration, Supervision, Writing – original draft, Writing – review & editing. JK: Data curation, Formal analysis, Project administration, Supervision, Writing – review & editing. RNas: Supervision, Writing – original draft, Writing – review & editing. SN: Conceptualization, Supervision, Writing – original draft, Writing – review & editing. RNaz: Writing – original draft, Writing – review & editing. HM: Data curation, Formal analysis, Writing – original draft, Writing – review & editing. CNu: Writing – review & editing. RK: Supervision, Writing – review & editing. RNan: Data curation, Formal analysis, Writing – review & editing. CNa: Data curation, Writing – review & editing. BNal: Supervision, Writing – review & editing. IN: Supervision, Writing – review & editing. MN: Supervision, Writing – review & editing. CK: Supervision, Writing – review & editing. ON: Supervision, Writing – review & editing. JN: Supervision, Writing – review & editing. BNam: Supervision, Writing – review & editing. PK: Project administration, Supervision, Writing – original draft, Writing – review & editing. GW: Supervision, Writing – review & editing, Formal analysis.
